# Supplementary material for: A high-density crowd state judgment model based on entropy theory
Source: PLoS One. 2021 Sep 2;16(9):e0255468. doi: 10.1371/journal.pone.0255468 (PMC8412320; doi:10.1371/journal.pone.0255468)
Supplement: Attachment — Submitted filename: Response to Reviewers.docx [file pone.0255468.s003.docx]

Dear reviewers:

Thank you for your letter and for the reviewers’ comments concerning our manuscript entitled “A high-density crowd state judgment model based on entropy theory” (ID: PONE-D-20-31256). Those comments are all valuable and very helpful for revising and improving our paper, as well as the important guiding significance to our researches. We have studied comments carefully and have made correction which we hope meet with approval. The revised parts have been marked out in the paper. The main corrections in the paper and the responds to the reviewer’s comments are as following:
Responds to the reviewer’s comments:

Reviewer #1:

1.Response to comment: In abstract the results should be included and discussed simultaneously.

Response: We have made correction according to the Reviewer’s comments. The research results are supplemented in the abstract. Because this paper focuses on the model, the correctness and practicability of the model are mainly described in the abstract.

2.Response to comment: The contribution of this work must be clearly describe in introduction section.

Response: As for the problems you mentioned, we have revised the introduction. The contribution of this paper is that, by further analyzing the shortcomings of previous studies, it is found that few scholars apply entropy theory to study the behavior of high-density people. The model of high-density crowd state judgment based on entropy theory established in this paper can make up for this deficiency and promote the study of entropy theory and crowd behavior.

3.Response to comment: Figure 1 is confusing make a proper diagram or flow chart which can highlight all states and clearly describe the direction or flow of each state.

Response: We are very sorry for the error in Figure 1 in the article. We have changed Figure 1 and adjusted the position of Figure 1 according to your suggestion, so we hope to get your approval.

4. Response to comment: Page 14 of 19 correct the statement crowd disturbance.

Response: We are very sorry for our incorrect writing. We have amended the term" the statement crowd disturbance" uniformly to " crowed state of confusions". It also corresponds to Figure 1.

5. Response to comment: Is it possible the models used in Figure 4 could describe as well.

Response: The model shown in Figure 4 is to analyze the relationship between population density and population density, so as to better study the change process of population status. As for your suggestion, we have carefully modified the analysis of the research results of the model in Figure 4 and hope to get your approval.

6. Response to comment: Where is the Green Guide Chart is there any reference or computation table available if yes please mention. “Table 3: Data flow calculated using the Green Guide chart”

Response: We are very sorry to have confused you. We have modified the title in Table 3. Crowd flow is calculated using the method given in reference [16-17] based on crowd flow = crowd density * crowd velocity, hence, the title of Table 3 is Data flow using the Green Guide. Data are selected from the Ando model. The Ando model is the study of population speed and density under the condition of high density of population, which is suitable for this paper.

7. Response to comment: A new section of results discussion must be added.

Response: According to your suggestion, we have supplemented the discussion at the front of the conclusion section and revised the conclusion.

Reviewer #2:

1.Response to comment: In the abstract section, the author/s have mentioned to $S_{0}$>0 (line 6), is it assumption, if not from where he got it.

Response: We are deeply sorry for our mistake. $S_{0}$ refers to entropy in the initial stat and *S* should refer to other stages, which we have modified. Entropy represents the degree of chaos of an event. The initial state is stable. With the development of an event, the greater the degree of chaos, the greater the entropy.

2.Response to comment: In section 2, High density crowd and state personnel, the author mentioned that both parameters involved in this model; the safety critical density of a stationary population and the safety critical density of a moving population are 4.7 and 4 persons/m^2. My suggestion is that they should have named the method or technique used in Ref. [11] to calculate these parameters.

Response: The continuous pedestrian flow model in the reference [11] you mentioned is built through the mathematical relationship between pedestrian speed and crowd density. This paper is a model to judge crowd state through entropy theory. As for the naming problem you raised, we have modified the unit of population density to pedestrians/m^2^ (ped/m^2^). Hope to get your approval.

3.Response to comment: In their study focused on investigating the proposed model in the industrial countries as a special case. The general formula which can be used to calculate all significant parameters involved in future outlook related to the same problem.

Response: Your suggestion is very important and will be a new direction for our future research.

4.Response to comment: In Figure 3, caption must have more explanation by specifying each color representing the type of pressure causing crowdy crowd (from low to high). Figure's 3 caption is not clear enough for the reader.

Response: We gratefully appreciate for your valuable suggestion. We have modified the title of Figure 3 and the graphic analysis. The pressure from the outer ring to the inner ring changes from small to large, with the minimum pressure at the edge and the highest pressure at the center.

I tried our best to improve the manuscript and made some changes in the manuscript. I appreciate for Editors warm work earnestly, and hope that the correction will meet with approval.

Once again, thank you very much for your comments and suggestion.

Best regards,

Falong He
