# Supplementary material for: A high-density crowd state judgment model based on entropy theory
Source: PLoS One. 2021 Sep 2;16(9):e0255468. doi: 10.1371/journal.pone.0255468 (PMC8412320; doi:10.1371/journal.pone.0255468)
Supplement: Attachment — Submitted filename: Response to Reviewers.docx [file pone.0255468.s004.docx]

Dear reviewers:

Thank you for your letter and for the reviewers’ comments concerning our manuscript entitled “A high-density crowd state judgment model based on entropy theory” (ID: PONE-D-20-31256). Those comments are all valuable and very helpful for revising and improving our paper, as well as the important guiding significance to our researches. We have studied comments carefully and have made correction which we hope meet with approval. The revised parts have been marked out in the paper. The main corrections in the paper and the responds to the reviewer’s comments are as following:
Responds to the reviewer’s comments:

Reviewer #3:

1.Response to comment: The written style of this paper should be revised to make it widely readerable.

Response: We have made correction according to the Reviewer’s comments. We have modified it according to the comments of reviewers. Some modifications have been made in the format and language of the paper, which we hope will make the paper more readable.

2.Response to comment: The authors established a high-density crowd state judgment model based on entropy theory, I suggest the authors to give more details on how to applied their model to deal real-world problems.

Response: As for the application of the model you mentioned, we have mainly supplemented it in the chapter of case analysis. In real-world problems, the model can be combined with the warning system to prevent the occurrence of crowd crowding events. According to the change of crowd status, the warning level will be divided into three levels: red alert, orange alert and yellow alert. Different levels of early warning take different emergency rescue measures to ensure the safety of the people.

3.Response to comment: some sentences are unreaderable, the authors should carefully revised their paper. For example: "Negative entropy flow obtained by a system from the outside world can reduce the total entropy, so that the system to the orderly direction of development, the system to maintain a stable state."

Response: We are very sorry that some of the sentences in the article are difficult for you to understand. We have revised the example sentences and some of the language in the article, hoping to make the article more readable and more accurate.

We tried our best to improve the manuscript and made some changes in the manuscript. We appreciate for Editors warm work earnestly, and hope that the correction will meet with approval.

Once again, thank you very much for your comments and suggestion.

Best regards,

Falong He
