# Supplementary material for: A high-density crowd state judgment model based on entropy theory
Source: PLoS One. 2021 Sep 2;16(9):e0255468. doi: 10.1371/journal.pone.0255468 (PMC8412320; doi:10.1371/journal.pone.0255468)
Supplement: Attachment — Submitted filename: Response to Reviewers.docx [file pone.0255468.s005.docx]

Dear reviewers:

Thank you for your letter and for the reviewers’ comments concerning our manuscript entitled “A high-density crowd state judgment model based on entropy theory” (ID: PONE-D-20-31256). Those comments are all valuable and very helpful for revising and improving our paper, as well as the important guiding significance to our researches. We have studied comments carefully and have made correction which we hope meet with approval. The revised parts have been marked out in the paper. The main corrections in the paper and the responds to the reviewer’s comments are as following:
Responds to the reviewer’s comments:

Reviewer #1:

1.Response to comment: The authors tried our best to improve the manuscript and made some changes in the manuscript. I accept the paper for publication in the previous round of review. While still i am suggesting to accept the paper for publication.

Response: Thank you very much for accepting my article.

Reviewer #4:

1.Response to comment: The biggest caveat it that it does not distinguish between what is already know in literature and want are the new results produced by the authors. Is it that the literature focuses mainly on density <= 4 ped/m2 and that the authors focus on higher density? If so, it should be stated clearly.

Response: 4p/m^2^ is the safety critical density of the movement group. This paper studies the prediction model of the crowded events in high density state with density greater than 4p/m^2^. Therefore, by analyzing and summarizing the previous studies of scholars and combining with the change of pedestrian speed, the state of pedestrians is divided to achieve the purpose of preventing dangerous events.

2.Response to comment: All the formulas in the text: where do they come from? how were they derived?

Response: Some well-known entropy formulas, no references.  The rest is basically the author's mathematical derivation, for such formulas, the author has a detailed written expression.

3.Response to comment: How were the numbers in tables and in text (ex: in section "Case Analysis") derived?

Response: The numbers in Table 3 are calculated according to the Green Guide research conclusions and the Ando model. Table 4 is based on Fig 3 and Table 3. In the "Case Study" section, the numbers are calculated by assuming the radius of the study area and by combining the mathematical formulas in the model with the range of population conditions. The remaining numbers in this paper are mainly obtained by summarizing and analyzing the references noted.

4.Response to comment: p. 21: what is the unit for S?

Response: Entropy in this paper is non-thermodynamic entropy, which is a value representing the disorder degree of an object without a clear unit.

5.Response to comment: What methods were used in the study?

Response: It has been clearly stated in the abstract that this paper adopts the thermodynamic entropy theory and the local equilibrium hypothesis. By analyzing and summarizing the previous studies on velocity and density as well as mathematical derivation, the entropy judgment model is established. The research methods are statistical analysis and mathematical derivation.

6.Response to comment: The authors claim for instance that "All these results are consistent with the actual situation (p. 21) ", "The results show that the models are effective" (Abstract); but nowhere is there a proof of such a claim. In other words: was the model validated?

Response: In response to your suggestion, we have added a chapter of “Model validation”. The simulation results show that there is a relationship between the crowd density, the crowd area in different conditions and the crowd system entropy. In addition, in the “Case analysis”, the calculation process of the model is clearly expressed for the convenience of readers. The results of case analysis and model verification are the same, which further proves the reliability and effectiveness of the model.

7.Response to comment: The list of the 4 types (free flow, maximum flow, intermittent flow, crowded state of confusions) is shown at 2 different places (p. 9 and p. 16)

Response: The four types of lists (free flow, maximum flow, intermittent flow, crowded state of confusions) displayed in two different places have different meanings. The former explains the four crowd states mainly studied in this paper, while the latter explains the speed, density and entropy corresponding to the four crowd states in detail.

8.Response to comment: There is a lot of typos on words (ex: "willimpact" instead of "will impact"), on formulas (ex: S?S2 instead of S>S2).

Response: We are very sorry that some of the sentences in the article are difficult for you to understand. We have revised the example sentences and some of the language in the article, hoping to make the article more readable and more accurate.

We tried our best to improve the manuscript and made some changes in the manuscript. We appreciate for Editors warm work earnestly, and hope that the correction will meet with approval.

Once again, thank you very much for your comments and suggestion.

Best regards,

Falong He
